# Biomechanical Analysis and Clinical Study of Augmented Versus Conventional Endoscopic Orbital Decompression for Dysthyroid Optic Neuropathy

**DOI:** 10.3390/bioengineering12060618

**Published:** 2025-06-05

**Authors:** Pengsen Wu, Yiheng Wu, Jing Rao, Shenglan Yang, Hongyi Yao, Qingjiang Liu, Yuqing Wu, Shengli Mi, Guiqin Liu

**Affiliations:** 1 Shenzhen Eye Hospital, Shenzhen Eye Medical Center, Southern Medical University, Shenzhen 518040, China; wups@mail2.sysu.edu.cn (P.W.); ykraojing@163.com (J.R.); yshlan328@126.com (S.Y.); danyuhou@163.com (Y.W.); 2 Bio-Manufacturing Engineering Laboratory, Tsinghua Shenzhen International Graduate School, Tsinghua University, Shenzhen 518055, China; wyh22@tsinghua.org.cn (Y.W.); yaohy19@mails.tsinghua.edu.cn (H.Y.); liuqj24@mails.tsinghua.edu.cn (Q.L.)

**Keywords:** finite element analysis, thyroid eye disease, dysthyroid optic neuropathy, biomechanics, orbital decompression surgery

## Abstract

Dysthyroid optic neuropathy (DON) represents a severe ocular complication in thyroid eye disease (TED) that can lead to vision loss. Although surgical decompression is a well-established treatment modality, the optimal decompression area remains controversial in orbital decompression surgery. **Purpose:** This study aims to develop and validate a finite element analysis (FEA) model of DON to compare the biomechanical behavior between patients undergoing conventional or augmented orbital decompression surgery, with potential clinical implications for surgical planning. **Methods:** FEA models were established using magnetic resonance imaging data from patients with myopathic TED. Pre-disease, preoperative, and postoperative FEA models were developed for both the conventional orbital decompression group and the augmented group, in which the posteromedial floor and the orbital process of the palatine bone were additionally removed to analyze the stress distribution and displacement of the optic nerve, eyeball, and orbital wall. A retrospective analysis was performed to validate the biomechanical analysis results. **Results:** The FEA results reveal that DON patients experience higher stress on the optic nerve, eyeball, and orbital wall than healthy individuals, mainly concentrated at the orbital apex. Postoperatively, the stress on the optic nerve was significantly reduced in both groups. In addition, postoperative stress on the optic nerve was significantly lower in the augmented group than in the conventional group. The clinical results demonstrate that patients in the augmented group experienced significantly faster and more pronounced improvements in visual acuity and visual field. **Conclusions:** FEA shows that augmented orbital decompression surgery can alleviate stress more effectively, especially for the optic nerve, which was validated by clinical analysis. This developed FEA model of DON may facilitate determining the appropriate surgical procedure for orbital decompression.

## 1. Introduction

Thyroid eye disease (TED), also known as Graves’ orbitopathy, is an organ-specific autoimmune inflammatory disorder experienced by patients with thyroid disease. Dysthyroid optic neuropathy (DON) is a potentially blinding complication that reportedly affects 5% to 8% of TED patients [1]. The pathogenesis underlying DON has been described as follows: direct compression of the optic nerve by enlarged extraocular muscles (EOMs) [2,3], stretching of the optic nerve by proptosis [4,5], alteration in orbital blood flow [6], and inflammation [7]. Timely evaluation and intervention are critical to avert irreversible visual impairment.

Based on the latest clinical practice guidelines, surgical decompression is recommended for DON, especially when treatment with steroids has been ineffective [8]. By performing orbital decompression surgery with one-, two-, or three-wall resection, particularly in the apical region, additional space is created to alleviate the compressive pressure on the optic nerve and improve its blood supply [1,9,10]. In recent years, a new technique for performing endoscopic orbital decompression in cases of DON has been proposed that involves the removal of the posteromedial floor and the orbital process of palatine bone (OPPB) in addition to the conventional inferomedial orbital wall decompression to achieve maximal orbital apex decompression. Postoperatively, the patients experience good visual function recovery with few complications [11,12]. Notwithstanding numerous modifications to the orbital decompression technique introduced over the years [13,14], there is still a lack of consensus on the optimal decompression area in orbital decompression surgery. Inadequate decompression at the orbital apex may result in poor vision improvement after surgery. Repeated orbital decompression surgery in such cases is technically challenging due to the presence of scars and the loss of clear anatomical landmarks [15,16]. Therefore, it is essential to establish quantitative indicators to guide preoperative decision-making regarding the location and extent of orbital decompression surgery.

Finite element analysis (FEA) is a crucial numerical technique in biomechanical research for predicting and optimizing the mechanical behavior of biological tissues, implants, and surgical procedures [17]. FEA has been utilized to simulate physiological processes such as ocular motility, elucidating the localized mechanical effects on critical orbital structures, including the optic nerve and posterior ocular tissues [18]. By simulating various mechanical loading conditions, FEA provides a computational assessment of the biomechanical behavior of internal fixation implants and fractured orbital bone segments, thereby assisting in the selection of optimal implant materials and surgical techniques [19,20]. For TED, FEA has been applied to examine the correlation between post-decompression volume changes and backward eyeball displacement, assessing surgical efficacy for exophthalmos reduction [21]. Moreover, FEA is a valuable tool for evaluating the stress distribution in the orbital wall and comparing postoperative tissue mechanics across different surgical techniques. This facilitates the selection of procedures and helps reduce damage to vital orbital structures, such as the EOMs [22,23]. However, current FEA applications for TED face several limitations. The technical challenges involved in measuring in vivo intraorbital pressure make it difficult to establish patient-specific biomechanical models. As a result, existing methods are unable to reliably predict postoperative stress distribution and tissue displacement patterns, which significantly restricts their predictive capability for surgical outcomes during preoperative planning.

This study aimed to develop an FEA model of DON to reveal the stress distribution within the orbits of DON patients. We compared the biomechanical behavior of the optic nerve, eyeball, EOMs, and orbital wall among patients who underwent different types of orbital decompression surgeries. Additionally, we conducted a retrospective analysis of visual function improvement in DON patients who underwent conventional versus augmented orbital decompression surgeries to validate the results of the biomechanical analysis.

## 2. Materials and Methods

### 2.1. MRI Data Collection

This retrospective clinical study was approved by the Ethics Committee of Shenzhen Eye Hospital (2024KYYJ150) and was performed in accordance with the Declaration of Helsinki. Written informed consent was obtained from all participants.

Twenty-two patients who underwent endoscopic transnasal orbital decompression surgery refractory to corticosteroid therapy at Shenzhen Eye Hospital from June 2018 to July 2024 were recruited. DON was diagnosed by evaluating visual field defects, lowered visual acuity, diminished color vision, or relative afferent pupillary defects, while ruling out other possible causes of optic neuropathy [9]. The baseline characteristics of the DON patients are summarized in Table A1. There were no significant differences between groups in age, sex, duration of thyroid disease, etc. Although the augmented group had a slightly higher clinical activity score (CAS) than the conventional group, this difference did not reach statistical significance (*p* = 0.09).

In addition, 40 patients (20 males and 20 females) with unilateral orbital trauma or tumors were included as normal controls (NCs), and MRI images of their unaffected side were used for modeling. The general clinical data of these patients are listed in Table A2.

3.0T MRI of the orbits was performed, covering the region from the supraorbital margin to the inferior border of the maxillary sinus. The imaging parameters were as follows: echo time (TE) = 2.46 ms, repetition time (TR) = 6.41 ms, resolution = 0.7 mm × 0.7 mm, and slice thickness = 1 mm.

### 2.2. Geometry and Finite Element Model

**Figure 1:**
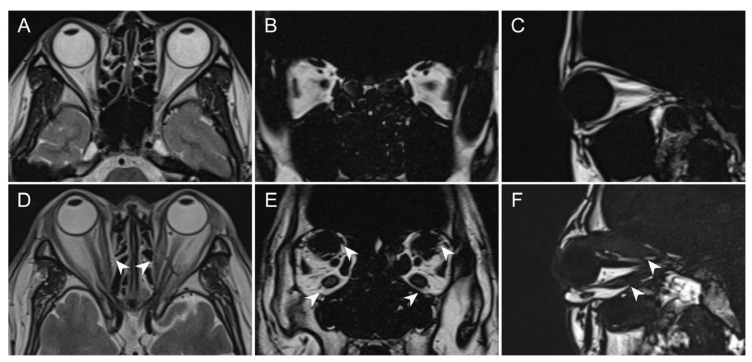
MRI findings comparing a healthy control and a DON patient. (**A**–**C**) Axial, coronal, and sagittal MRI images demonstrate normal orbital anatomy in the control subject. (**D**–**F**) Significant EOM enlargement is observed in all imaging planes of a DON patient (white arrowhead).

**Figure 2:**
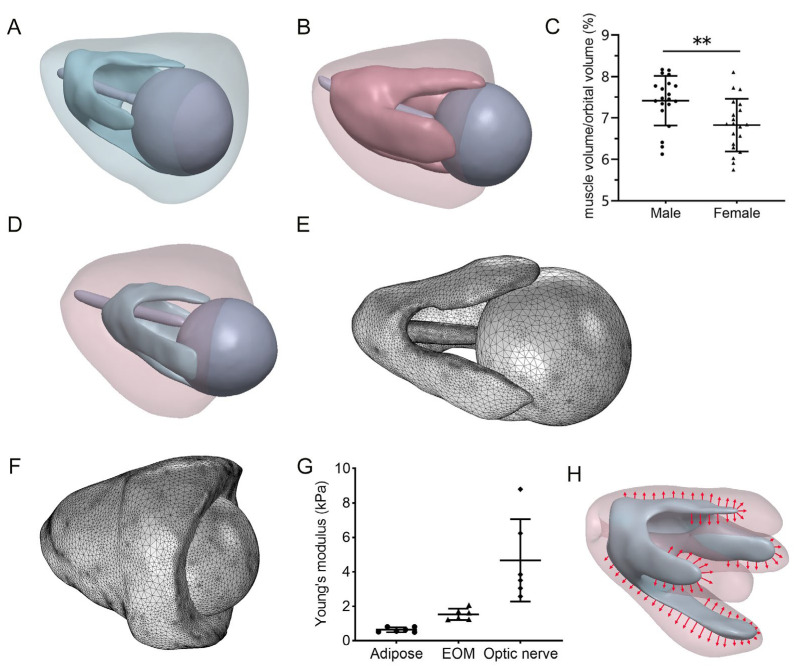
FEA model establishment and simulation. Comparative 3D reconstructions of orbital anatomy in (**A**) a healthy control versus (**B**) a DON patient, demonstrating characteristic EOM enlargement. (**C**) The calculated ratio of EOM volume to orbital volume in healthy individuals. Statistical analysis was performed using an unpaired *t*-test. ** *p* < 0.01. (**D**) The pre-disease FEA model for a DON patient. (**E**) Integrated model including the EOMs, eyeball, and optic nerve. (**F**) Three-dimensional orbital model. (**G**) Measured Young’s modulus of orbital adipose tissue, EOM, and optic nerve from six normal control individuals. (**H**) The progression of TED was simulated by expanding the EOM in the FEA model. Inside is the pre-disease EOM model, and the semi-transparent outer layer represents the current diseased EOM model.

A comprehensive three-dimensional (3D) orbit model was constructed, including the orbital wall, adipose tissue, EOMs, optic nerve, and eyeball, using MRI images from DON patients as well as normal control (NC) subjects (Figure 1). The MRI images were imported into Mimics 21.0 software (Materialise, Leuven, Belgium) in DICOM format. The geometries of the bony orbit, EOMs, eyeball, and optic nerve were reconstructed by defining their boundaries through grayscale thresholding, using semi-automated segmentation of MRI scans. The remaining orbital volume was modeled as adipose tissue. The volumes of both the EOMs and the orbital bony cavity were then quantitatively analyzed (Figure 2A,B). The ratio of the EOM volume to the orbital volume in healthy individuals was calculated (Figure 2C).

A major innovation of this model is the development of a pre-disease orbital FEA model for patients with TED. Based on the characteristics of myopathic TED, the primary change observed in patients following disease onset is the thickening of the EOMs, whereas other orbital components (adipose tissue, optic nerve, and globe) remain unchanged [24]. The pre-disease EOM volume was calculated by applying the normative EOM-to-orbit-volume ratio (from NC subjects) to each patient’s measured orbital volume (Equation (1)). Therefore, utilizing this orbital model for TED patients allows us to construct a pre-disease orbital FEA model specific to each patient (Figure 2D).

(1) $$\mathrm{Pre-disease}\;\mathrm{EOM}\;\mathrm{volume}=\mathrm{orbital}\;\mathrm{volume}\;\mathrm{of}\;\mathrm{TED}\;\mathrm{patient}\;\times \;\frac{EOM\;volume\;of\;NC}{Orbital\;volume\;of\;NC}$$

Further model optimization was performed with Geomagic Studio 12.0 (Geomagic Inc., Cary, NC, USA), including hole repair, irregular part correction, and surface refinement. The 3D model assembly was conducted using SOLIDWORKS 2024 (SolidWorks Corp., Waltham, MA, USA) based on the relative position and connection between each part.

The assembled 3D model was imported into COMSOL Multiphysics 6.3 (COMSOL Inc., Burlington, MA, USA) for FEA. All elements were modeled as explicit tetrahedra with mesh sizes ranging from 0.19 to 2.56 mm to ensure computational accuracy while maintaining result consistency. The final mesh configuration comprised 114,433 nodes, 640,648 tetrahedral elements, and 80,139 triangular elements. The complete anatomical assembly, including the EOMs, eyeball, and optic nerve, is presented in Figure 2E, while the corresponding 3D orbital model is shown in Figure 2F. The mesh density was validated numerically by conducting a convergence test.

### 2.3. Material Properties

To optimize computational efficiency and reduce processing time, this study adopted a simplified modeling approach based on recent studies [25,26,27]. The optic nerve, adipose tissue, eyeball, and EOMs were modeled as linear elastic, isotropic, and homogeneous materials, characterized by two key coefficients that define their mechanical properties: Young’s modulus (E) and Poisson’s ratio (ν) [23]. The Young’s modulus of the eyeball was obtained from previous experimental measurements and finite element analyses [28].

The elastic modulus of the EOMs, adipose tissue, and optic nerve was measured using an atomic force microscope (AFM). For Young’s modulus, the optic nerve was measured as an integrated structure, encompassing both the nerve sheath and the axon bundle. These tissues were obtained from 6 patients who underwent enucleation surgery. The characteristics of these patients are summarized in Table A3. Briefly, the experiments were conducted using an AFM (Nanowizard ULTRA Speed 2, JPK Instruments, Berlin, Germany). Data were obtained in Quantitative Imaging mode (QI mode) utilizing PFQNM-LC cantilevers, which have a nominal spring constant of 0.25 N/m and a conical tip with a 10 nm radius (Bruker, Santa Barbara, CA, USA). Before each experiment, the cantilever spring constant and sensitivity were calibrated using the thermal fluctuation method, producing spring constant values from 0.05 to 0.018 N/m. The sample was fixed to the base of a 3 cm cell culture dish and maintained in a hydrated state by applying PBS droplets. The cell culture dish was then fixed on the AFM sample stage. Afterward, the AFM tip was engaged in the center of the sample to scan an area measuring 1.2 μm × 1.2 μm. A force trigger of 2 nN was applied to the cantilever tip, with a Z ramp of 2 μm at a speed of 2 μm/s. Force curves were acquired via contact-mode force mapping and analyzed using JPK Data Processing software (Bruker, Santa Barbara, CA, USA; Version spm-8.0.144). Young’s modulus was calculated by applying the Hertzian contact model to the retraction segment of the force curves. The results are shown in Figure 2G.

Consistent with previous reported finite element models, we assumed that the eyeball exhibits partial compressibility with a Poisson’s ratio of ν = 0.4, whereas the optic nerve, adipose tissue, and EOMs were modeled as nearly incompressible with a Poisson’s ratio of ν = 0.49 (Table 1) [25,26,27].

### 2.4. Finite Element Model Simulation

Tissue contact interactions were modeled based on methods established in previous studies [25,29]. The optic nerve was geometrically integrated at the posterior pole of the globe, with interfacial bonding between all components achieved through identity pairs to form a unified assembly. For boundary conditions, the orbital wall was fixed in all degrees of freedom, while the other components were free to move. The expansion of the EOMs was simulated by applying a specific displacement to the outer surface of the EOMs in an outward perpendicular direction.

For myopathic TED, the essential pathological change is the enlargement of the EOMs, with relatively minor changes in the adipose tissue [24]. We expanded the pre-disease EOM model to the current diseased EOM volume to simulate the progression of TED (Figure 2H). The muscle expansion was modeled indirectly through thermal expansion using COMSOL. Specifically, a temperature field was applied to the muscle region, and the material’s thermal expansion coefficient was defined to regulate volumetric changes, effectively achieving a simulated “expansion” of the muscle. For NCs, physiological muscle pretension is present throughout the musculoskeletal system. In the absence of experimental data on EOMs, we applied a uniform pretension of 80 mN inward and perpendicular to the muscle surface for each rectus muscle in our model, consistent with established loading parameters from prior computational studies [28]. A comprehensive FEA was performed to evaluate the von Mises stress distribution, calculate the surface-average stress, and assess the displacement within the orbital contents.

### 2.5. Orbital Decompression Surgery and Clinical Data Collection

The participants were divided into two groups, the conventional group and the augmented group, according to the surgical procedures they accepted. Endoscopic endonasal spheno-ethmoidectomy procedures were performed. In brief, after the exposure and removal of the orbital floor and medial wall, for conventional orbital decompression surgery, the medial wall was accurately removed up to the area where the medial minor wing of the sphenoid bone intersects with the optic canal near the orbital apex, thoroughly exposing the annulus of Zinn. For the augmented group, in addition to encompassing the full extent of conventional surgery, both the OPPB and the maxillary bone lateral to the OPPB were also removed until the maxillary sinus posterior and lateral walls met. Then, the standard annulus of Zinn incision, periorbita incision, and removal of orbital fat were performed as previously described [10].

Follow-up visits were scheduled at 3 days, 1 week, 1 month, and 3 months post-surgery, during which the best corrected visual acuity (BCVA) was assessed. At the one-month follow-up, additional Humphrey visual field tests (HFA 860, Carl Zeiss Meditec Inc., Dublin, CA, USA) were conducted, with measurements taken both before and after surgery.

### 2.6. Statistical Analysis

Statistical analysis and figure drawing were conducted using GraphPad Prism (Prism 8, GraphPad Software, San Diego, CA, USA). The results are presented as mean ± standard deviation (SD). For the analysis of continuous data between the two groups, the student’s *t*-test was employed for normally distributed data, while the Mann–Whitney test was utilized for non-normally distributed data. Fisher’s exact test was selected to analyze the categorical data. To compare multiple groups, one-way ANOVA or the Kruskal–Wallis test was used, depending on the normality of the distribution of the data. Statistical significance was defined as *p* < 0.05.

## 3. Results

### 3.1. Increased Intraorbital Pressure in DON Patients

**Figure 3:**
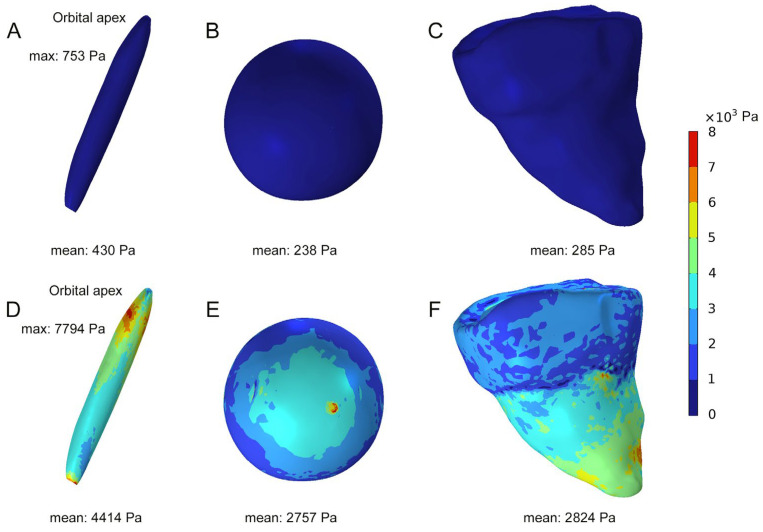
Comparison of the stress distribution within the orbits between a healthy individual and a DON patient using FEA models. (**A**–**C**) The stress distribution on the optic nerve, eyeball, and orbital wall of a healthy individual. (**D**–**F**) The stress distribution on the optic nerve, eyeball, and orbital wall of a patient with DON.

Currently, there is very limited data on the intraorbital pressure in healthy individuals. In this study, the stress distribution on the orbital soft tissues in a healthy individual was analyzed using an FEA model. The results indicated the mean stresses were 430 Pa on the optic nerve, 238 Pa on the eyeball, and 285 Pa on the orbital wall (Figure 3A–C). By simulating the EOM enlargement in DON patients, we established an FEA model of DON and performed biomechanical analysis. The results demonstrated that the mean stresses were 4414 Pa on the optic nerve, 2757 Pa on the eyeball, and 2824 Pa on the orbital wall, all significantly higher than normative values in healthy controls. In addition, the stress distribution on the optic nerve is uneven in this DON case, with a peak stress of 7794 Pa concentrated at the orbital apex (Figure 3D–F).

### 3.2. Pronounced Pressure Reduction Following Augmented Orbital Decompression Surgery

**Figure 4:**
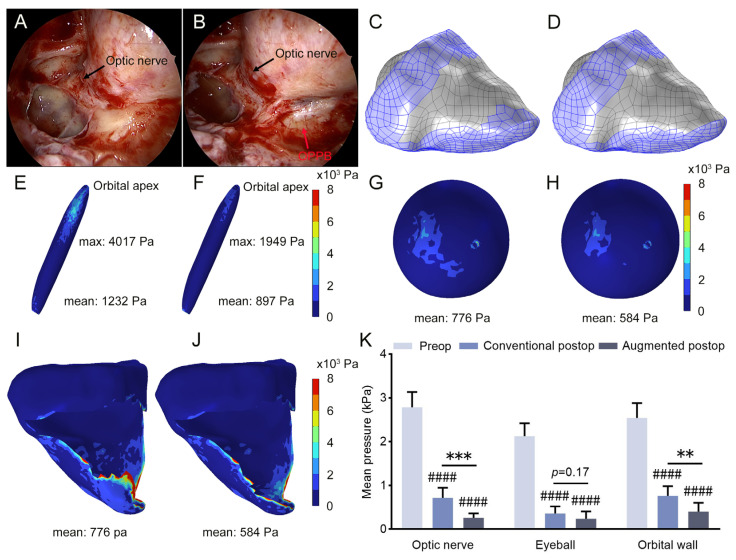
Comparison of stress distribution within the orbits in postoperative FEA models of patients undergoing conventional versus augmented orbital decompression surgery. (**A**,**B**) Photos of the orbital apex during endoscopic orbital decompression surgery. Compared to conventional orbital decompression surgery (**A**), the bony structures of the orbital process of palatine bone (OPPB) and the maxillary bone lateral to the OPPB were additionally removed in the augmented surgery (**B**), as indicated by the red arrow. (**C**,**D**) Postoperative FEA models of the conventional and augmented groups were developed by removing the constraints of the orbital bone on the TED model, in accordance with the extent of the orbital decompression surgery. (**E**–**J**) The FEA models illustrate the postoperative stress distribution on the optic nerve, eyeball, and orbital wall following the conventional or augmented orbital decompression surgery. Left: conventional group, right: augmented group. (**K**) Statistical analysis of the mean stress on the optic nerve, eyeball, and orbital wall was performed using one-way ANOVA. Preop, preoperation; Postop, postoperation. #### *p* < 0.0001 compared with preoperative levels, ** *p* < 0.01, *** *p* < 0.001.

In this study, postoperative FEA models were constructed based on the location and extent of orbital decompression (Figure 4A–D). Biomechanical analysis was then performed, and the results revealed that postoperative stresses on the optic nerve, eyeball, and orbital wall were reduced in both groups compared to preoperative levels. However, comparing the postoperative results between these two groups, the stress on the optic nerve at the orbital apex remained higher in the conventional group (Figure 4E–J). In addition, we established FEA models and performed biomechanical analysis using MRI data from 11 DON patients (11 eyes). Statistical analysis revealed that the mean stresses on the optic nerve, eyeball, and orbital wall were significantly reduced after operation in both groups. Nonetheless, postoperative analysis revealed significantly higher mechanical stress on the optic nerve and orbital wall in the conventional approach than in the augmented approach. No significant difference in the postoperative stress on the eyeball was observed between these two groups (Figure 4K). The FEA results demonstrated that augmented orbital decompression surgery provides better decompression outcomes.

### 3.3. Prediction of Postoperative Soft Tissue Displacement

**Figure 5:**
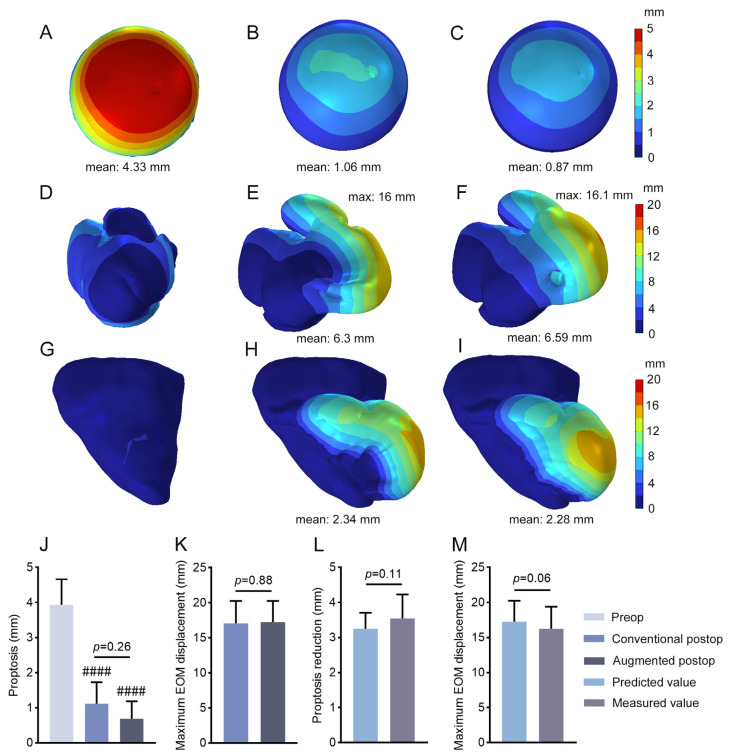
Comparison of displacement of the eyeball, EOMs, and adipose tissue. (**A**–**I**) The displacement of the eyeball, EOMs, and adipose tissue was simulated before surgery, following conventional orbital decompression surgery or augmented surgery, respectively. (**J**) Comparison of proptosis between preoperative levels and the FEA-model-predicted postoperative levels in the conventional or augmented group was conducted using one-way ANOVA. (**K**) Comparison of the FEA-model-predicted maximum EOM displacement between postoperative levels in the conventional or augmented group was performed using a paired *t*-test. (**L**,**M**) Comparison of predicted values obtained from the FEA model with the measured reductions in proptosis and maximum EOM displacement was analyzed using a paired *t*-test. Preop, preoperation; Postop, postoperation. #### *p* < 0.0001 compared with preoperative levels.

The postoperative displacement of the eyeball, EOMs, and adipose tissue was analyzed using this FEA model, with the results showing that, compared to the preoperative state, proptosis reduction along with the displacement of the EOMs and adipose tissue toward the adjacent paranasal sinus were observed in both groups after surgery (Figure 5A–I). Statistical analysis indicates a significant reduction in proptosis in both groups postoperatively. However, the difference in postoperative proptosis of these two cohorts is not statistically significant (Figure 5J). In addition, the differences in the maximum displacement of EOM are not statistically significant between these two groups (Figure 5K). Furthermore, we compared the predicted values obtained from the FEA model with the measured reductions in proptosis and EOM displacement. The results showed no statistically significant difference between the predicted and measured values (Figure 5L,M).

### 3.4. More Effective Visual Function Improvement Following Augmented Orbital Decompression Surgery

**Figure 6:**
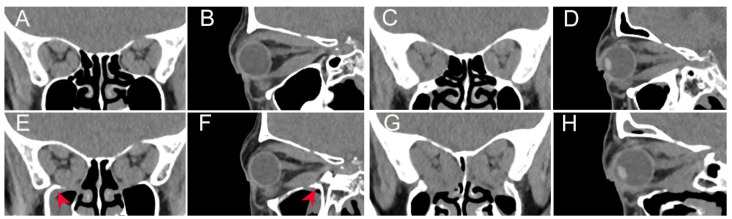
Orbital CT scans reveal that augmented orbital decompression surgery provides better decompression at the orbital apex. (**A**,**B**) Preoperative CT scans of a DON patient who underwent conventional orbital decompression surgery. (**C**,**D**) Preoperative CT scans of a DON patient who underwent augmented orbital decompression surgery. (**E**,**F**) Postoperative CT scans of the patient who underwent conventional orbital decompression surgery. (**G**,**H**) Postoperative CT scans of the patient who underwent augmented surgery. The arrows highlight the area of the orbital process of palatine bone (OPPB) that has been preserved following conventional orbital decompression surgery.

Preoperative CT scans show enlargement of the EOMs, crowding at the orbital apex, and compression of the optic nerve in DON patients (Figure 6A–D). Postoperative CT scans reveal that the orbital cavity has expanded in both groups. Compared to the postoperative CT scans in the conventional group (Figure 6E,F), the additional removal of the OPPB area led to a more extensive orbital decompression in the augmented group (Figure 6G,H).

**Figure 7:**
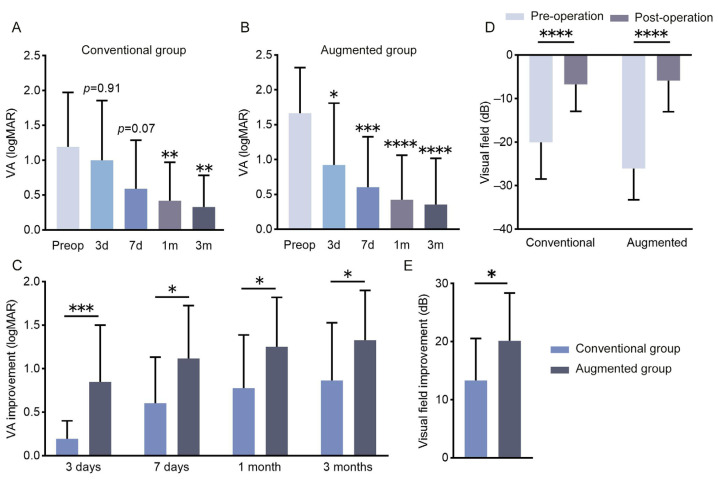
DON patients undergoing augmented orbital decompression surgery experienced better improvement in visual acuity and visual field defect. (**A**,**B**) Comparison of postoperative visual acuity at 3 days, 7 days, 1 month, and 3 months with preoperative levels. Statistical analysis was conducted using one-way ANOVA. (**C**) Comparison of postoperative visual acuity improvement between the conventional and augmented groups at different follow-ups. Intergroup comparisons at postoperative day 3, day 7, and month 3 were performed using unpaired *t*-tests, while the Mann–Whitney U test was employed for comparisons at 1 month postoperatively. (**D**) Visual field outcomes at one month postoperatively were compared with preoperative levels within each group using the Mann–Whitney U test. (**E**) Intergroup comparison of visual field improvement was analyzed with unpaired *t*-test. VA, visual acuity; Preop, preoperation; d, day; m, month. * *p* < 0.05, ** *p* < 0.01, *** *p* < 0.001, **** *p* < 0.0001.

The clinical results show that postoperative visual acuity (VA) improved in both groups compared to before surgery. Comparing early postoperative VA of these two groups with preoperative levels, at 3 days and 1 week after surgery, VA improved significantly in the augmented group, while the conventional group had no significant change (Figure 7A,B). Comparing the VA improvement between the two groups, the results demonstrate that the augmented group experienced significantly better improvements in VA at several postoperative intervals (Figure 7C). At one month postoperation, both groups show significant improvement in visual field defects, with patients in the augmented group experiencing greater improvement (Figure 7D,E).

Preoperatively, diplopia was present in four members (36.36%) of the conventional group and three members (27.27%) of the augmented group. Postoperatively at one month, there were three new-onset cases in the conventional group and four in the augmented group, with no significant difference between the groups. No patients in either group experienced complications such as cerebrospinal fluid leak or severe bleeding.

## 4. Discussion

Based on research findings on the varying effects of TED on orbital fat and EOMs, TED is categorized into two subtypes. Approximately 2/3 of TED patients are characterized by adipose tissue proliferation [30,31], while 1/3 of TED patients have a myopathic phenotype with normal adipose tissue [24]. This study primarily investigated the biomechanical alterations in myopathic TED. The expansion of EOMs results in secondary soft tissue congestion and optic nerve compression, ultimately leading to DON. DON is the most severe complication of TED, and the biomechanics of the orbital contents play a critical role in its pathogenesis. However, to date, there have been only a limited number of TED biomechanics studies reported [21,22,23], and there have been no reports on finite element models specifically addressing DON.

### 4.1. Prediction of Mechanical Stress Distribution Within the Orbit for DON

Increased intraorbital pressure is a hallmark clinical feature of TED [2]. The key pathogeneses in TED include inflammation, adipogenesis, and glycosaminoglycan production, leading to an enlargement of the orbital contents. Because the orbital tissues are encased in a bony funnel, an increase in orbital soft tissue volume raises the intraorbital pressure. This can result in venous outflow obstruction, elevated IOP, and orbital content protrusion [32]. In addition, the optic nerve and other intraorbital nerve branches may be damaged by direct elevated pressure. Although intraorbital pressure is critically important in TED, it remains particularly challenging to measure. Historically, the preferred approach for estimating intraorbital pressure involved assessing tissue resistance triggered by the eyeball’s retropulsion [33]. However, this approach has certain limitations, as it can only measure the pressure in the anterior part of the orbit, and the accuracy of the measurement is relatively poor. Currently, although direct measurement of intraorbital pressure is technically possible through various experimental methods, it remains impractical in clinical environments due to its complexity, invasiveness, and limited availability. In addition, the orbit is segmented into multiple sub-compartments by muscles and septa. Thus, the clinical application of direct pressure measurements may be limited because intraorbital pressure can differ in various parts of the orbit [33]. This study presents a new method for investigating the stress distribution in the orbit by simulating the pathological process of EOM expansion in TED. The calculated mean stress on the optic nerve is 4414 Pa, with a maximum stress of 7794 Pa in DON patients, which is generally consistent with the experimentally measured retrobulbar pressures of 28.7 mmHg (range 17–40 mmHg) reported in the literature [34,35]. Intraorbital pressure in DON patients is significantly higher than in healthy individuals, which is estimated to be 3–6 mmHg [36]. This FEA enables the non-invasive estimation of the stress distribution within the orbit.

### 4.2. Prediction of Postoperative Stress Distribution Within the Orbit

The generally accepted mechanism for DON suggests that enlarged EOMs compress the apical optic nerve, leading to axonal damage [37]. Histopathological research has shown that axonal loss is more evident in the apical section of the optic nerve [1]. This study demonstrates that in DON patients, the stress distribution on the optic nerve is uneven, with the highest stress concentrated at the orbital apex, reaching up to 7794 Pa. To reduce optic nerve damage and restore visual function, adequate decompression of the orbital apex is necessary for patients with DON undergoing orbital decompression surgery [11,12,38]. In this study, the augmented orbital decompression surgery additionally removed the posteromedial floor and the OPPB. The clinical outcomes demonstrate that, compared to the conventional group, patients in the augmented group experienced faster and better visual function improvement.

The enlargement of EOMs is the key factor leading to DON [2,3,39]. However, the degree of EOM enlargement varies among patients with TED, with some individuals experiencing enlargement of one or more EOMs [40]. A volumetric analysis indicates that the enlargement of the medial rectus muscle primarily contributes to DON [3], while other studies demonstrate that the significant factor for DON is the enlargement of the superior levator and superior rectus complex or the superior oblique muscle [40,41]. In different TED cases, the enlarged EOM varies, the degree of EOM enlargement differs, and the orbital geometry exhibits notable differences [3,42]. Thus, varying distributions of stress within the orbit can be observed. Currently, various orbital decompression techniques are employed for DON, including two-wall decompression (inferior and lateral), three-wall decompression (inferior, lateral, and medial), and endoscopic endonasal inferomedial wall decompression, etc. [43]. Although numerous studies are being conducted regarding orbital decompression, consensus on the optimal method has not been reached [44]. This study offers a novel approach for selecting the optimal orbital decompression surgery using an FEA model. The stress distribution within the orbit can then be described. In addition, by simulating the surgical procedure of bony wall removal, a postoperative FEA model can be constructed to assess the alleviation of optic nerve compression. This FEA model facilitates determining the optimal decompression location and extent for the surgical treatment of DON.

### 4.3. Prediction of Postoperative Complications and Exophthalmos Reduction

In addition to restoring visual function, orbital decompression surgery addresses exophthalmos, preventing exposure keratopathy while improving aesthetic appearance. This patient-specific FEA model facilitates the precise quantification of globe displacement, enabling the comprehensive assessment of disease progression in pathological exophthalmos and the predictive evaluation of postoperative exophthalmos reduction outcomes. Moreover, following orbital decompression surgery, the EOMs may migrate toward adjacent sinuses. This displacement is a recognized contributor to postoperative diplopia, with the degree of EOM protrusion into the sinus cavity correlating positively with diplopia risk [45]. This study proposes a new method to predict the position of the EOMs after orbital decompression surgery using FEA, which has the potential to assess the occurrence of postoperative diplopia.

Compared to the reported FEA models of TED [21,22,23], the FEA model proposed in this study has the following advantages: it utilizes patient-specific MRI data for modeling, along with measured biomechanical parameters, resulting in a more personalized model. By expanding the EOMs, it simulates the pathological process of thickening in EOMs for numerical analysis, exploring stress distribution and displacement. Furthermore, it proposes the simulation of postoperative FEA models based on the location and extent of orbital decompression surgery, analyzing the stress distribution of important structures such as the optic nerve, as well as the displacement of the eyeball and EOMs, providing objective and quantitative references for the choice of surgical methods.

This study has certain limitations. This FEA model is constructed by simulating the process of EOM enlargement and is unsuitable for patients with lipogenic TED, which is characterized by adipose proliferation without EOM enlargement. In addition, due to the lack of a reliable intraorbital pressure measurement method, the stress predictions generated by this FEA model cannot be directly validated. Furthermore, modeling orbital tissues as linearly elastic materials has inherent limitations. The progression of TED can lead to the remodeling of orbital connective tissue and EOMs, which alters their biomechanical properties. Due to the varying disease severity among different TED patients, the biomechanical characteristics of the adipose and muscles may differ.

Future work will focus on simulating the process of fat proliferation in patients with lipogenic TED to develop an FEA model tailored to the biomechanical analysis needs of various TED types. Additionally, by incorporating direct measurements of orbital pressure obtained during orbital decompression surgery after removing the orbital wall, we will further validate the accuracy of the stress distribution in the proposed FEA model and make necessary adjustments to enhance its precision. Furthermore, future studies should focus on measuring the elastic modulus of the orbital tissues in TED patients with varying degrees of fibrosis, employing more suitable material modeling methods, and developing personalized FEA models that incorporate patient-specific biomechanical properties of orbital soft tissues to improve simulation accuracy.

## 5. Conclusions

In summary, this study introduces a novel approach for investigating the stress distribution in the orbit using an FEA model of patients with DON, and with the simulation of the orbital decompression surgery, the postoperative stress distribution and orbital soft tissue displacement can be analyzed. In addition, the clinical data indicate that the augmented orbital decompression surgery resulted in faster and better visual function improvement, validating the biomechanical analysis results. This developed FEA model can provide valuable insights for selecting the optimal orbital decompression surgery for DON patients.

## Figures and Tables

**Table 1:** Tissue biomechanical properties used for the model.

Tissue	Constitutive Model	Biomechanical Properties	Source
Optic nerve	Isotropic elastic	Elastic modulus = 4663 Pa	Experimentally determined
		Poisson’s ratio = 0.49	Liu et al. [27]
Adipose tissue	Isotropic elastic	Elastic modulus = 636.5 Pa	Experimentally determined
		Poisson’s ratio = 0.49	Liu et al. [27]
Eyeball	Isotropic elastic	Elastic modulus = 500 KPa	Schutte et al. [25]
		Poisson’s ratio = 0.4	Schutte et al. [25]
EOMs	Isotropic elastic	Elastic modulus = 1529 Pa	Experimentally determined
		Poisson’s ratio = 0.49	Wang et al. [26]

## Data Availability

The numerical model and raw files used in this research are available at SimTK https://simtk.org/projects/don20250516/#, accessed on 16 May 2025.

## Abbreviations

DON: dysthyroid optic neuropathy; FEA: finite element analysis; MRI: magnetic resonance imaging; TED: thyroid eye disease; EOM: extraocular muscle; OPPB: orbital process of palatine bone; AFM: atomic force microscope; CAS: clinical activity score; BCVA: best corrected visual acuity; VEP: visual evoked potential; CT: computed tomography; SD: standard deviation; VA: visual acuity; IOP: intraocular pressure.

## Appendix A

**Table A1:** Demographic data of patients with DON undergoing conventional or augmented orbital decompression surgery.

	Conventional Group	Augmented Group	*p*-Value
No. of participants	11	11	
No. of eyes	18	16	
Sex, male/female	6/5	3/8	0.39 ^a^
Age, years	52.36 ± 7.87 (39–64)	56.36 ± 10.5 (38–70)	0.32 ^b^
CAS	3.9 ± 1.22 (3–6)	4.9 ± 1.14 (3–6)	0.09 ^c^
Duration of thyroid disease, months	18 ± 11.36 (6–39)	16.09 ± 12.49 (5–45)	0.66 ^c^
Duration of TED, months	13.45 ± 11.64 (3–39)	11 ± 8.28 (4–33)	0.88 ^c^
Duration of VA impairment, months	5 ± 2.9 (1–10)	6.23 ± 4.7 (0.5–17)	0.47 ^b^
Hyperthyroid	2 (18.19%)	1 (9.09%)	>0.99 ^a^
Hypothyroid	1 (9.09%)	1 (9.09%)	>0.99 ^a^
Euthyroid	8 (72.73%)	9 (81.82%)	>0.99 ^a^
History of I131 treatment	2 (18.19%)	1 (9.09%)	>0.99 ^a^
History of radiotherapy	1 (9.09%)	3 (27.27%)	0.59 ^a^
History of glucocorticoid pulse therapy	5 (45.45%)	10 (90.9%)	0.06 ^a^

CAS: clinical activity score; TED: thyroid eye disease; VA: visual acuity. ^a^ Fisher’s exact test; ^b^ Unpaired Student’s *t*-test; ^c^ Mann–Whitney U test.

**Table A2:** Demographic data of patients with unilateral orbital disease.

	Male	Female	*p*-Value
No. of participants	20	20	
Age, years	45.1 ± 12.75 (18–63)	46.35 ± 10.45 (25–71)	0.74 ^a^
Unilateral orbital tumor	16 (80%)	16 (80%)	>0.99 ^b^
Unilateral ocular tumor	3 (15%)	2 (10%)	>0.99 ^b^
Unilateral orbital trauma	1 (5%)	2 (10%)	>0.99 ^b^

^a^ Unpaired Student’s *t*-test; ^b^ Fisher’s exact test.

**Table A3:** Demographic data of patients undergoing enucleation surgery.

Patient ID	Age (Years)	Gender	OD/OS	Diagnosis
Patient 1	19	Male	OS	Phthisis bulbi
Patient 2	49	Female	OS	Corneal ulcer
Patient 3	29	Male	OD	Corneal staphyloma
Patient 4	51	Male	OD	Bullous keratopathy
Patient 5	25	Female	OS	Corneal staphyloma
Patient 6	68	Female	OD	Corneal ulcer
